# Intestinal Tissue Damage Reduction After Distal Perfusion for Aortic Arch Surgery in a Neonatal Porcine Model

**DOI:** 10.3390/biomedicines14020355

**Published:** 2026-02-03

**Authors:** Kristin Klaeske, Sabine Meier, Jana Lammers, Susann Ossmann, Mia Bovet, Michael A. Borger, Maja-Theresa Dieterlen, Martin Kostelka, Marcel Vollroth

**Affiliations:** Department of Cardiac Surgery, Helios Clinic, University of Leipzig, Heart Center Leipzig, 04289 Leipzig, Germany

**Keywords:** intestine, neonates, pediatric, aortic arch surgery, cardiopulmonary bypass, selective anterograde cerebral perfusion

## Abstract

**Background**: Aortic arch reconstruction in neonates is often challenging, owning its surgical complexity and postoperative complication risk. To assess intestinal damage, we compared selective anterograde cerebral perfusion (SACP) and SACP with additional distal perfusion (SACP + DP) used in aortic arch surgery in a neonatal piglet model. **Methods**: Piglets underwent cardiac arrest for 60 min with SACP (*n* = 9) or SACP + DP (*n* = 9), followed by a 120 min recovery. Hemodynamic parameters, blood gases and electrolytes were monitored. Biopsies of the small intestine and colon were analyzed for histopathological changes, intestinal barrier function, and oxidative stress. **Results**: Hemodynamic measurements and electrolyte concentrations were comparable between SACP and SACP + DP (*p* > 0.05), except for potassium levels during cardiac arrest (*p* = 0.03). Blood lactate levels (*p* < 0.01) were elevated and pH values (*p* < 0.01) were reduced in the SACP group during cardiac arrest. Morphometric analysis of the intestinal tissue revealed longer crypts (*p* = 0.02) and a thicker mucosal layer (*p* = 0.05) of colonic structures in the SACP group. Compared to SACP, the mRNA expression of cytoprotective Parkinson’s disease protein DJ-1 (*p* = 0.02) and hypoxia-inducible nuclear factor erythroid 2-related factor 2 (*p* = 0.04) were higher in the small intestine of the SACP + DP group. The marker of epithelial barrier function, E-cadherin, showed lower mRNA expression in the colon of the SACP + DP group (*p* = 0.02). **Conclusions**: Our study results showed that SACP + DP revealed less intestinal tissue damage and loss of structural integrity, as well as an upregulation of cytoprotective molecules and anti-oxidative stress mechanisms. Therefore, SACP + DP is a reliable procedure in our model for aortic arch surgery that can contribute to better postoperative outcomes by reducing intestinal damage.

## 1. Introduction

Aortic arch reconstruction in neonates is often challenging due to postoperative complications, which involves not only cardiac but also abdominal organ dysfunction [1,2].

The gastrointestinal tract is one of the most vulnerable organs, where reduced perfusion and ischemia-reperfusion (IR) injury can result in serious complications such as vascular injury, necrotizing enterocolitis and gastrointestinal (GI) bleeding after cardiac surgery [3,4,5,6]. GI bleeding affects 0.2–2% of pediatric patients and is associated with high mortality rates (8.8–19%) [3].

The optimal cannulation strategy during aortic arch surgery in neonates remains controversial and depends on several factors such as patient characteristics, vascular anatomy, surgical procedure complexity, intraoperative settings and duration of reconstruction [7,8]. Currently, the concept of moderate-to-mild hypothermia (≥28 °C) with selective anterograde cerebral perfusion (SACP) is an effective and widely used method of cardiopulmonary bypass (CPB) for cerebral protection during aortic arch reconstruction [9,10]. During SACP, collateral vessels arising from the subclavian and intercostal arteries provide limited blood flow to the lower body and abdominal organs [1]. However, this collateral perfusion accounts for only about 5% of the total CPB flow, which is substantially lower than the physiological gastrointestinal perfusion of approximately 16% of cardiac output [11]. As a result, marked hypoperfusion of the abdominal organs may occur, leading to IR injury and a higher incidence of intestinal complications with increased postoperative morbidity and mortality [12,13].

Therefore, alternative perfusion strategies have been introduced. A previous study showed that a combined cerebral and visceral perfusion strategy provides better renal flow, hepatic function and tissue perfusion when compared with anterograde cerebral perfusion during aortic arch reconstruction in neonates [14]. Further, it was reported that SACP with additional distal perfusion (SACP + DP) was beneficial for patients in terms of a shorter ICU stay and a lower degree of acute kidney injury [7]. A prospective study by Soynov et al. showed that aortic arch reconstruction in neonates using the method of full-body perfusion reduces the incidence of neurobiological lesions and renal complications requiring renal replacement therapy [15]. Moreover, whole-body perfusion led to fewer intraoperative blood products and coagulation factor transfusion compared to SACP alone in neonatal aortic arch surgery [16]. The administration of distal perfusion systems has been shown to be beneficial not only in relation to CPB in neonates. In patients undergoing femoral veno-arterial extracorporal membrane oxygenation cannulation, distal perfusion catheter placement might be associated with a decreased risk of vascular complications and limb ischemia [17]. More recently, our research group demonstrated that SACP + DP has nephroprotective effects compared to SACP in a neonate piglet model [18]. However, the molecular effects resulting from different perfusion strategies on intestinal mucosal damage remain unclear.

Intestinal mucosal damage can be evaluated through morphometric analyses in combination with the improved Chiu’s score grading according to microscopic evidence of histopathological changes [19]. At the cellular level, tight junctions and adherens junctions such as E-cadherin are crucial structures for maintaining epithelial integrity [20]. Furthermore, various intracellular signaling processes, including cytoprotective mediators and transcription factors, have been shown to counteract oxidative injury as a consequence of peripheral hypoperfusion [21].

Based on these factors, this study aimed to compare whether the effectiveness of SACP + DP is superior to SACP regarding intestinal damage and oxidative stress in a neonatal piglet model for aortic arch surgery.

## 2. Materials and Methods

### 2.1. Animals and Anesthesia Management

For this study, one- to four-week-old, suckling German Landrace/hybrid with Peitrain piglets (2.5–10 kg) of both sexes were randomly assigned to the treatment groups SACP (*n* = 9) and SACP + DP (*n* = 9). Before transportation, piglets were sedated with midazolam (Hameln Pharma GmbH, Hameln, Germany) 0.5–1 mg/kg, atropine (B.Braun SE, Melsungen, Germany) 0.02 mg/kg, and ketamine (Serumwerk Bernburg AG, Bernburg, Germany) 22 mg/kg intramuscular. Anaesthesia was maintained by intravenous application of 8–16 mg/kg/h propofol (Fresenius Kabi Deutschland GmbH, Bad Homburg, Germany) and 35 µg/kg/h fentanyl (Dechra Veterinary Products Deutschland GmbH, Aulendorf, Germany). Detailed information about anesthesia management were summarized in the Appendix A.

### 2.2. Surgical Technique, Extracorporeal Circulation Strategy, and Perfusion

Piglets were placed in a supine position. Following median full sternotomy, thymic tissue was excised, and the pericardium was opened. For better exposure, stay sutures at the edges of the pericardium were placed.

Identification and meticulous preparation of the anatomical structures including the aorta and pulmonary trunk, the innominate artery, and the right atrium were performed using electrocautery. Subsequently, 50 Prolene-sutures were placed in the innominate artery and the right atrial appendage allowing cannulation for CPB. For arterial cannulation, a 2.6 mm cannula (Sorin Group, Mirandola, Italy) was used. Venous cannulation was achieved by placing a 12F 90–angled cannula (free life medical GmbH, Aachen, Germany) in the right atrium. After achieving an activated clotting time (ACT) > 400 s, CPB (Stöckert S5, LivaNova, Munich, Germany) with a calculated flow depending on body surface area and cardiac index was initiated. The heart-lung machine starts with a calculated flow of 3.0 L/m^2^ body surface area (175–200 mL/kg). The body surface area of the pig is calculated as follows: square root of (body length × body weight/3600). Head perfusion should account for approximately one third of the calculated body flow and occur at a mean arterial pressure of 50 mmHg [22].

In the SACP + DP group, an additional 6F cannula (Terumo Europe NV, Leuven, Belgium) was inserted via the femoral artery using Seldinger technique, positioned in the area of the visceral vessels and connected to a roller pump, integrated into the CPB system. This most commonly used clinical technique is easier and safer compared to surgical cannulation of the thoracic aorta. Then, “crash cooling” was initiated with the help of a hypothermia device (Tianjin Welcome Medical Equipment Co., Tianjin, China) (temperature setting 15 °C) to a body target temperature of 28 °C and controlled by nasal and rectal temperature monitoring sites. During hypothermia, the settings of the hypothemia device were adapted to 28 °C. After reaching a body core temperature of 28 °C, the aorta was cross-clamped, and the heart was arrested using a single dose of 35 mL/kg bodyweight, cold-modified del Nido cardioplegia [23] administered in the aortic root through a 4F pediatric aortic root cannula (Medtronic, Minneapolis, MN, USA). The cerebral vessels were snared for selective anterograde perfusion with a CPB flow of 30%. In the piglets assigned for SACP and distal perfusion (SACP + DP), additional perfusion of the lower body with 35% of the estimated flow was commenced through the distal perfusion cannula in the femoral artery.

After a 60 min cardiac arrest at 28 °C, the aortic clamp was removed, the cerebral vessels were released, selective distal perfusion was stopped, and CPB flow was increased to the full estimated flow to facilitate reperfusion and warming in full body perfusion. The rewarming rate during CPB is typically around 0.2 °C/min—no more than 0.5 °C/min.

**Figure 1:**
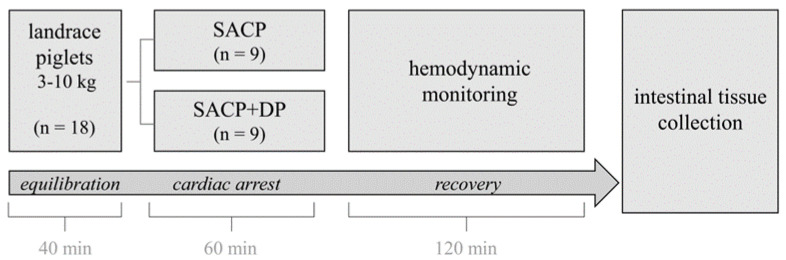
The 40 min equilibration period was defined as the time between induction of anesthesia and aorta cross-clamping. Piglets were randomly assigned to receive either selective anterograde cerebral perfusion (SACP, *n* = 9) or SACP with additional distal perfusion (SACP + DP, *n* = 9) during a 60 min cardiac arrest. The aortic clamp was removed, followed by approximately 45 min of reperfusion and weaning from CPB. After a total recovery time of 120 min, intestinal tissue and blood were collected. Reproduced with the acceptance of [18].

After hypothermia, body temperature is raised to normal within 30 min during the reperfusion phase (temperature setting of hypothermia device 39 °C). On average, the cooling and rewarming phases ranged between 10 and 15 min. Thereafter, the animals were weaned from CPB within a time frame of 15 min followed by recovery. Volume and catecholamine were administered to keep the mean arterial blood pressure above 50 mmHg. After a 120 min recovery (including reperfusion), the piglets were euthanized, and tissue samples were obtained. The study design is illustrated in Figure 1.

### 2.3. Preparation of Specimens

Intestinal biopsies from the small intestines at the distal end of the ileum and the colon were extracted and then paraffin-embedded for histological analyses or snap-frozen for molecular analyses.

Detailed information about blood and tissue sampling as well as further molecular biological and protein biochemical analyses were summarized in the Appendix A.

### 2.4. Histological Measurement of Intestinal Tissue

**Figure A1:**
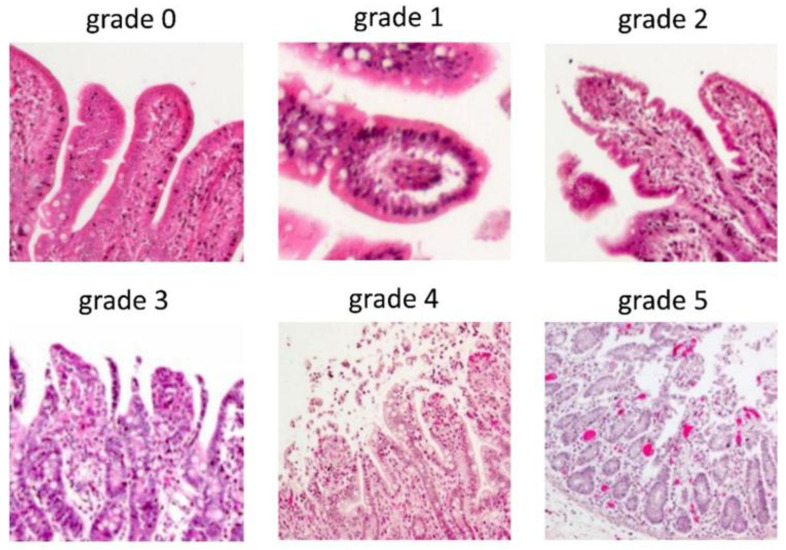
Representative image of hematoxylin/eosin G-stained small intestinal cross sections illustrating progression of ischemic intestinal mucosal lesions, graded from 0 to 5 (Chiu score).

**Figure A2:**
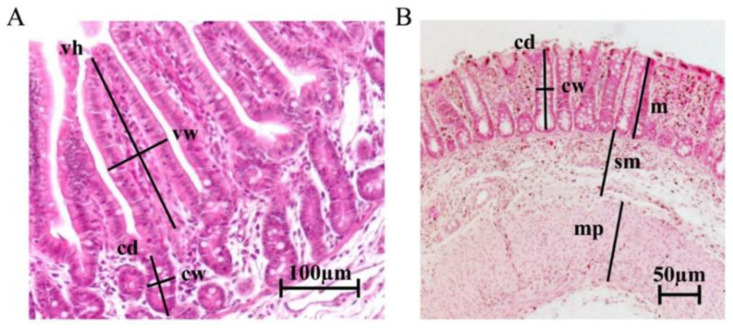
Morphometric analysis. (**A**) Representative image of hematoxylin/eosin G-stained small intestinal cross sections and exemplary measurement of villus height (vh), villus width (vw), crypt depth (cd), and crypt width (cw). (**B**) Representative image of hematoxylin/eosin G-stained colonic cross sections and exemplary measurement of crypt depth (cd), crypt width (cw), and thickness of mucosa (m), submucosa (sm), and muscularis propria (mp).

Hematoxylin/Eosin G staining of paraffin-embedded intestinal tissue was performed on 3 µm sections as described previously [24]. Intestinal mucosal damage of the small intestine was evaluated using the improved Chiu’s score according to intestinal epithelial disruption and changes of the villus structure [19]. The score was defined by the following criteria: grade 0, normal mucosal villus structure; grade 1, development of subepithelial Gruenhagen’s space, usually at the tip of the villus; grade 2, extension of the subepithelial space with moderate epithelial detachment limited to the upper half of the villus; grade 3, massive epithelial lifting towards the crypt with isolated denudation of the villus tips; grade 4, frequent villi denudations with dilated capillaries tissue; and grade 5, disintegration of the lamina propria, ulceration, hemorrhage and loss of villus tissue. Higher scores were interpreted as indicating more severe damage [21]. Multiple observers were involved in the assessment to validate scoring consistency. Representative images for Chiu score criteria were illustrated in Figure A1. Further morphometric analyses were performed by measuring villus height (from the tip to the base, excluding the intestinal crypt), villus width (measured halfway between the base and the tip), crypt depth (from the base upward to the region of transition between the crypt and villus), and crypt width (measured at the middle of each longitudinal crypt section) of the small intestine. Colonic sections were analyzed for crypt depth, crypt width, the height of enterocytes, and the thickness of mucosa, submucosa, and muscularis propria [25]. Twenty measurements were taken per sample for the morphometric analyses of the intestinal tissue. Mucosa, submucosa, and muscularis propria thickness was measured six times per colon sample. Representative images for morphometric measurements were illustrated in Figure A2. The histological evaluation was performed using the Axio Plan 2 microscope (Carl Zeiss AG, Jena, Germany) and AxioVision Release 4.8.2 SP3 software (Carl Zeiss AG) at the same magnification with the same microscopic settings for standardized image analysis.

### 2.5. Statistical Analysis

Statistical analyses were performed using SPSS Statistics 28 software (IBM, Armonk, New York, USA). Values were expressed as mean and 95% confidence interval (95% CI) unless stated otherwise. The Shapiro–Wilks test was used for checking normality of data. Two-group comparisons of means were performed with an unpaired independent t-test for normal distributed, metric parameters. The Mann–Whitney U test was used for non-parametric, non-normally distributed values. Pearson Chi-Square test was used for categorical data. The sample size was determined by a priori power analysis (G*Power 3.1; effect size f = 0.65, α = 0.05, power = 0.8), including the calculation of the effect size with the aid of published standard deviations for lactate as the primary endpoint, resulting in the sample size of nine piglets per group. *p* values ≤ 0.05 were considered statistically significant.

## 3. Results

### 3.1. Hemodynamic Measurement, Blood Gas Analyses and Oxygen Saturation

The study groups were comparable for hemodynamic parameters during equilibration, aorta cross-clamp, a 60 min ischemia and after a 120 min recovery (*p* > 0.05), except for central venous pressure during equilibration (SACP: 11.6 mmHg [95% CI: 4.9–18.2]; SACP + DP: 6.2 mmHg [95% CI: 5.5–7.0]; *p* = 0.05; Table 1).

Blood lactate levels increased above the reference range of 0.5–2.2 mmol/L in both groups after a 60 min cardiac arrest and remained high after a 120 min recovery. Lactate levels were higher in the SACP group (equilibration: *p* = 0.03, 60 min cardiac arrest: *p* < 0.01, 120 min recovery: *p* = 0.03) compared to the SACP + DP group. pH values differ after a 60 min cardiac arrest, where the pH value was lower in the SACP group compared to the SACP + DP group (*p* < 0.01). The SACP and SACP + DP groups had comparable electrolyte concentrations of blood sodium, chloride, and calcium at equilibration, aortic cross-clamp, a 60 min cardiac arrest, and after a 120 min recovery (all *p* > 0.05). After a 60 min cardiac arrest, the blood potassium was higher in the SACP group (5.83 mmol/L [95% CI: 4.88–6.79]) compared to the SACP + DP group (4.91 mmol/L [95% CI: 4.51–5.31]; *p* = 0.03) (Table 2).

Thoracic NIRS measurement demonstrated comparable levels of oxygen saturation during the equilibration period (SACP: 58% [95% CI: 53–64]; SACP + DP: 58% [95% CI: 53–64]; *p* = 0.45). Compared to SACP + DP (56% [95% CI: 48–63]), a reduction in thoracic NIRS was observed after aorta cross-clamp in the SACP group (40% [95% CI: 30–51]; *p* = 0.01) and persisted during the period of cardiac arrest (SACP: 37% [95% CI: 29–46]; SACP + DP: 56% [95% CI: 52–61]; *p* < 0.01). After 120 min of recovery, thoracic NIRS returned to comparable values between both groups (SACP: 44% [95% CI: 37–51]; SACP + DP: 51% [95% CI: 45–56]; *p* = 0.14).

### 3.2. Histopathological Assessment of Intestinal Tissue

Compared to SACP + DP, colonic tissue of the SACP group had elongated crypts (*p* = 0.02) and a thicker mucosa layer (*p* = 0.05) (Table 3). Villus height, villus width, crypt width and the thickness of submucosa, and muscularis propria were comparable between the SACP and SACP + DP group (*p* > 0.05).

### 3.3. Chiu Score Classification

The Chiu score classification of ischemic mucosal damage [19] of the small intestine revealed comparable scores in the SACP and SACP + DP group (*p* = 0.19) (Table 4). However, a higher grade of tissue erosion (*p* = 0.05) was documented in the small intestine of the SACP group compared to the SACP + DP group (Table 5).

### 3.4. Gene Expression of Hypoxia-Inducible Factors, Cytoprotective Protein, Cell Adhesion Junction, and Tight Junctions

Gene expression levels of cytoprotective Parkinson’s disease protein (DJ-1), nuclear transcription factors nuclear factor erythroid 2-related factor 2 (NRF2) and hypoxia-induced factor-1α (HIF-1α), tight junctions claudin-1 and occludin as well as adhesion junction E-cadherin were measured in the small intestine and colonic tissue. Primer sequences were listed in the Appendix A (Table A1). Compared with the SACP group, the mRNA expression of DJ-1 and NRF2 were increased in small intestinal tissue of the SACP + DP group (*p* < 0.05) (Table 6). The mRNA levels of E-cadherin in the colonic tissue decreased markedly in the SACP + DP group when compared to the SACP group (*p* = 0.04).

### 3.5. Oxidative Stress in Intestinal Tissue

Oxidative stress was quantified by measuring enzyme activities of nicotinamide-adenine dinucleotide phosphate oxidase (NOX), superoxide dismutase (SOD), and catalase. In the small intestine and the colon, the enzyme activities of NOX, SOD and catalase were comparable between the SACP and SACP + DP group (Table 7).

Protein expression of the oxidative stress sensor DJ-1 was comparable between the SACP and the SACP + DP group in the small intestine (SACP: 288 pg/mg [95% CI: 222–356]; SACP + DP: 248 pg/mg [95% CI: 161–334]; *p* = 0.20) and the colon (SACP: 316 pg/mg [95% CI: 196–436]; SACP + DP: 242 pg/mg [95% CI: 131–354]; *p* = 0.49). The protein expression of NRF2, an anti-oxidative transcription factor, was comparable between the SACP group (2.2 ng/mg [95% CI: 1.9–2.6]) and the SACP + DP group (2.7 ng/mg [95% CI: 2.0–3.5]; *p* = 0.09) in the small intestine. In the colonic tissue, a reduced NRF2 protein expression was detected in the SACP + DP group (2.7 ng/mg [95% CI: 1.9–3.6]) compared to the SACP group (3.6 ng/mg [95% CI: 2.9–4.3]; *p* = 0.05).

### 3.6. Protein Expression of E-Cadherin

**Figure A3:**
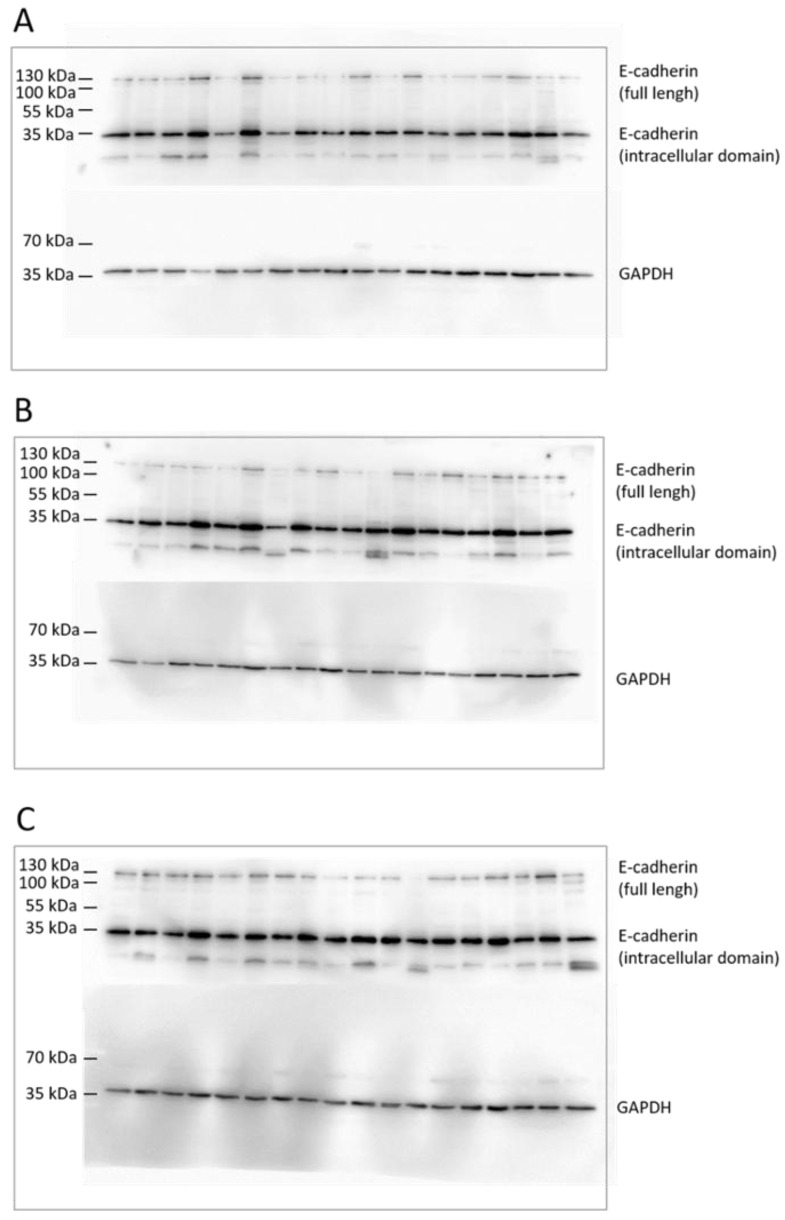
Protein expression of full-length E-cadherin (135 kDa) and the intracellular domain of E-cadherin (33 kDa) was determined by western blot analysis (**A**–**C**). The protein lysate (30 µg) from consecutive piglet samples was loaded with the small intestine and colon. Glyceraldehyde-3-phosphate dehydrogenase (GAPDH) was used as the loading control.

The protein expression of full-length E-cadherin and the intracellular domain of E-cadherin were used to estimate the structural integrity of the intestinal epithelium [20]. While full-length E-cadherin represents intact adhesion junctions, the intracellular domain of E-cadherin displays disrupted protein. Full-length E-cadherin protein levels of the SACP and SACP + DP group were comparable in the small intestine (SACP: 0.63 arb.U [95% CI: 0.31–0.95]; SACP + DP: 0.58 arb.U [95% CI: 0.40–0.76]; *p* = 0.39) and colon (SACP: 1.22 arb.U [95% CI: 0.11–2.33]; SACP + DP: 0.68 arb.U [95% CI: 0.48–0.88]; *p* = 0.67). The protein expression of intracellular E-cadherin showed comparable results between both groups in the small intestine (SACP: 3.83 arb.U [95% CI: 2.48–5.18]; SACP + DP: 2.79 arb.U [95% CI:2.31–3.26]; *p* = 0.14) and the colon (SACP: 5.63 arb.U [95% CI: 1.83–9.43]; SACP + DP: 3.91 arb.U [95% CI: 3.05–4.79]; *p* = 0.96) (Figure A3).

### 3.7. Apoptosis Induction in Intestinal Tissue

The release and activation of apoptosis-inducing factor (AIF) into the cytosol was comparable between the SACP and SACP + DP group in the intestine (SACP: 4.6 ng/mg [95% CI: 3.5–5.6]; SACP + DP: 5.1 ng/mg [95% CI: 3.3–6.8]; *p* = 0.28) and the colon (SACP: 6.3 ng/mg [95% CI: 5.2–7.5]; SACP + DP: 6.4 ng/mg [95% CI: 4.4–8.3]; *p* = 0.50). Cytosolic cytochrome c release did not differ between the groups in the small intestine (SACP: 0.06 ng/mg [95% CI: −0.01–0.13]; SACP + DP: 0.18 ng/mg [95% CI: 0.00–0.36]; *p* = 0.34) and colonic tissue (SACP: 0.19 ng/mg [95% CI: 0.06–0.31]; SACP + DP: 0.20 ng/mg [95% CI: −0.03–0.42]; *p* = 0.67).

## 4. Discussion

This study compared the impact of SACP and SACP + DP on intestinal tissue damage in a neonatal piglet model for aortic arch surgery. Our results demonstrated that SACP + DP induces less intestinal tissue damage and loss of structural integrity. In detail, the SACP group showed a higher degree of morphometric changes demonstrated by elongated crypts and thicker mucosa layers in the colon as well as more tissue erosions in the small intestine compared to the SACP + DP group. Chiu scoring revealed comparable ischemic mucosal damage between the two groups. Furthermore, SACP + DP showed a stronger induction of anti-oxidative and anti-apoptotic mechanisms indicated by increased mRNA expression of the cytoprotective protein DJ-1 and hypoxia-inducible transcription factor NRF2, along with a lower protein expression of cytosolic NRF2.

The intestinal mucosa is particularly sensitive to IR injury. A decrease in cardiac output and the altered blood circulation through collaterals during peripheral hypoperfusion in the SACP group could reduce the gastrointestinal perfusion leading to mucosal ischemia and the disruption of intestinal structures [26].

Hemodynamic parameters of the intraoperative monitoring were comparable in both groups. Blood concentrations of sodium, chloride, and calcium were comparable in our neonatal piglet model. Electrolyte imbalances could not be detected. These findings underline the stable experimental conditions. However, we documented higher potassium levels during cardiac arrest in the SACP group that could be associated with metabolic acidosis [27], which is consistent with our findings of a decreased pH in the SACP group during cardiac arrest. As a consequence of cardiac arrest, we documented the accumulation of blood lactate under hypoxic conditions in the SACP group. Lactate results from anaerobic metabolism can be used as a biomarker to estimate the function of intestinal epithelium because high lactate levels may indicate intestinal damage caused by tissue hypoperfusion and hypoxia [21,28].

In cardiac surgery, marked hypoperfusion of abdominal organs are often observed during and after CPB. The intestinal epithelium reacts particularly sensitive to any reductions in blood flow and oxygen supply, resulting in cellular oxidative stress, mitochondrial dysfunction and the subsequent induction of apoptosis [29]. Oxidative stress is evidenced by the generation of reactive oxygen species (ROS), which plays an important role in the early stages of intestinal injury [30]. In this study, the measurement of ROS-producing and ROS-degrading enzymes demonstrated comparable enzyme activities in the SACP and SACP + DP groups. Furthermore, mitochondrial damage due to oxidative stress is linked to the release of proapoptotic cytochrome c and AIF [31]. Our results did not detect differences in cytochrome c or AIF release. Therefore, it can be concluded that the cellular and mitochondrial stress is comparable between both perfusion strategies during CPB.

Furthermore, the duration and type of ischemia, the time of reperfusion, and the segment of the intestine play a crucial role in the severity of mucosal injury [29,32]. Previous studies have shown that the colon is less susceptible to ischemia-reperfusion-induced tissue injury than the small intestine, as demonstrated by less epithelial damage and apoptosis [33]. Intestinal mucosal damage can be evaluated through morphometric analysis in combination with the improved and widely used scoring system by Chiu et al. because it is easy to apply and reproduce [34]. During intestinal injury, the formation of Gruenhagen’s spaces at tip of a villus was attributed to the accumulation of cytoplasmic fluid from ischemic cells and was shown to occur within 30 min of ischemia [19]. After Chiu scoring, we detected pathological alterations to the intestinal tissue that were independent of the used perfusion strategy. In both study groups, more than 75% of piglets had a Chiu score ≥ grad 3. However, the Chiu score was comparable between SACP and SACP + DP. In addition, increased rates of tissue erosion in the small intestine indicated greater intestinal injury under hypoperfusion. Similar histopathological mucosa damage has previously been described in rodent IR models [21,32]. Although hyperemia and edema were comparable between the two groups, the higher grade of hyperemia in the SACP group after surgical intervention could be caused by a special form of hyperemia produced by congestion and discharge, which can occur after reperfusion of temporarily hypoperfused tissue. In cases of short-term increased blood flow, e.g., due to hemodynamic congestion and reperfusion in the SACP group, it is conceivable that under acute conditions and efficient drainage, the development of edema has not yet been observed at higher grades [35,36].

The morphological surface enlargement resulting from crypt elongation indicates an adaptation process to the injured colonic tissue in the SACP group, as known in short bowel syndrome [37]. The increased thickness of the *tunica mucosa* in colonic tissue has demonstrated an association with impaired nutrition adsorption and augmented higher energy consumption [25]. This may result in impaired tissue regeneration in the small intestine and the colon following hypoperfusion in the SACP group. Consequently, we suggest a more beneficial effect of the additional distal perfusion in our study cohort.

Under hypoxic conditions, cells at the villus tips in the small intestine and colonic surface epithelium initially lose their connection to the basement membrane with progressive loss of cells extending towards the crypt base with progressing ischemia [19,29]. Tight junctions and adherens junctions are crucial structures for the formation and maintenance of epithelial barrier function, and E-cadherin is one of the most important molecules that influences mucosal permeability and tissue repair [20]. Here, we detected a higher E-cadherin mRNA expression in colonic tissue in the SACP group, which may be explained by an upregulation of adhesion molecules due to peripheral hypoperfusion. However, this effect was not observed in the small intestine, suggesting an early adaptation process of the colon following IR-injury to protect epithelial barrier function. In general, the loss of E-cadherin expression results in reduced cell–cell adhesion and loss of structural integrity [38]. Previous studies have reported that E-cadherin is degraded during intestinal ischemia or internalized in response to inflammatory mediators [39,40]. In our study, the protein expression of both full-length and intracellular E-cadherin were comparable between the SACP and SACP + DP groups. This suggests that in our setting the E-cadherin protein expression was not affected by different perfusion methods.

DJ-1 is known as a cytoprotective protein regulating multiple cellular processes involved in the regulation of oxidative stress, apoptosis, and transcriptional signaling to promote cell survival and protect from myocardial injury following IR [21]. On the one hand, DJ-1 promotes the hypoxic response under oxygen depletion, and on the other hand, it protects against cell damage after reoxygenation [41]. In the SACP + DP group, the observed increase in DJ-1 mRNA expression may reflect a higher cytoprotective effect in the small intestine after hypoperfusion, although protein expression was comparable.

Further, DJ-1 can indirectly modulate gene expression by interacting with transcription factors like NRF2. NRF2 regulates the expression of antioxidant proteins, thus protecting against ROS-induced tissue damage [21,41]. Our results showed higher mRNA expression of NRF2 in the small intestine of the SACP + DP group, while the protein expression of NRF2 in colonic tissue was reduced in the SACP + DP group indicating the translocation of NRF2 into the nucleus. We suggest that the DJ-1/NRF2 signaling pathway is induced in the SACP + DP group, playing a crucial role in the protective response to oxidative stress conditions.

The finding that perfusion-induced changes in mRNA and protein levels differ between the small intestine and the colon emphasizes the impact of complex regulatory mechanisms in response to hypoperfusion and oxidative stress at multiple levels. Transcriptional regulation has been observed to occur predominantly in the small intestine, while the colon responds to oxidative stress at the translational level. This observation is consistent with the results of a previous study, which reported divergent regulations in mRNA and protein expression in different intestinal segments [42].

The observed comparable protein expressions of DJ-1 and E-cadherin may be attributable to the relatively short recovery period of 120 min or may be indicative of post-translational modifications or differences in protein stability.

Although clinical data are available, detailed analyses of intestinal effects depending on the perfusion strategy are currently not available due to protection of vulnerable pediatric patients. In this study, well-defined experimental data were collected to assess the considerable risks associated with the use of either selected cerebral perfusion or additional distal perfusion. The pig serves as a useful animal model for the study of pathophysiological conditions such as IR injury and clinical signs of disease relevant to the human GI tract [43]. Further, there is a correlation between human and pig, especially concerning the microvascular architecture [44]. Consequently, the anatomical and cardiovascular similarities between humans and pigs allow a careful translation implication of the findings, and we aim to integrate this protocol into routine clinical surgical practice.

However, there are some limitations. First, we used young and healthy piglets that lacked the risk factors, comorbidities, and comedications. Second, the surgical team could not be blinded with respect to the perfusion strategy that was used, which could introduce a bias. Third, we used surrogate parameters such as NRF2 and HIF1α to assess the molecular changes induced by changed oxygen conditions. Fourth, we do not have a control group for the evaluation under physiological conditions. Finally, the results are limited to a 120 min recovery.

## 5. Conclusions

In summary, the results of our neonatal piglet model showed that SACP + DP revealed less intestinal tissue damage and loss of structural integrity, as well as an upregulation of cytoprotective molecules and anti-oxidative stress mechanisms.

Together with the recently published results on nephroprotective effects in our neonate piglet model, the present study demonstrates that an additional distal perfusion strategy for abdominal organs is a reliable procedure that can contribute to better postoperative outcomes by reducing intestinal damage. It is therefore recommended that SACP + DP should be considered for implementation in routine clinical surgical practice.

## Figures and Tables

**Table 1:** Hemodynamic parameters in the SACP and SACP + DP group during equilibration, aorta cross-clamp, a 60 min cardiac arrest and after a 120 min recovery.

	SACP (*n* = 9)	SACP + DP (*n* = 9)	*p* Value
HR [bpm]			
equilibration	160 [125–196]	147 [124–170]	0.24
120 min recovery	150 [121–180]	158 [140–177]	0.31
CO [L/min]			
equilibration	1.15 [0.9–1.4]	1.63 [0.1–3.2]	0.25
aorta cross-clamp	2.17 [−1.0–5.3]	1.43 [−0.5–3.3]	0.32
60 min cardiac arrest	1.02 [−0.2–2.3]	2.35 [−0.1–5.2]	0.08
120 min recovery	0.87 [0.6–1.1]	1.52 [0.5–2.6]	0.09
MAP [mmHg]			
equilibration	57 [47–67]	65 [50–80]	0.17
aorta cross-clamp	45 [19–70]	45 [31–59]	0.50
60 min cardiac arrest	62 [24–101]	50 [41–59]	0.25
120 min recovery	48 [41–56]	61 [27–94]	0.21
CVP [mmHg]			
equilibration	11.6 [4.9–18.2]	6.2 [5.5–7.0]	0.05
aorta cross-clamp	6.4 [3.3–9.6]	6.7 [2.9–10.5]	0.46
60 min cardiac arrest	7.4 [3.8–11.1]	7.1 [0.1–14.1]	0.46
120 min recovery	11.4 [5.7–17.2]	7.0 [5.6–8.4]	0.06
RR_sys_ [mmHg]			
equilibration	77 [69–85]	79 [59–100]	0.39
aorta cross-clamp	49 [20–78]	49 [33–65]	0.49
60 min cardiac arrest	68 [27–110]	54 [43–65]	0.23
120 min recovery	73 [60–86]	77 [46–108]	0.40
RR_dias_ [mmHg]			
equilibration	47 [36–57]	53 [39–66]	0.21
aorta cross-clamp	40 [17–62]	42 [29–55]	0.42
60 min cardiac arrest	55 [22–88]	44 [39–50]	0.23
120 min recovery	37 [30–44]	52 [16–87]	0.18

Values shown as mean [95% confidence interval]. CO, cardiac output; CVP, central venous pressure; HR, heart rate; MAP, mean arterial pressure; RRsys/dias, systolic/diastolic blood pressure; SACP, selective anterograde cerebral perfusion; SACP + DP, selective anterograde cerebral perfusion with additional distal perfusion.

**Table 2:** Blood gas analysis and blood electrolyte concentration in the SACP and SACP + DP group during equilibration, at aortic cross-clamp, after a 60 min cardiac arrest and after 120 min of recovery.

	SACP (*n* = 9)	SACP + DP (*n* = 9)	*p* Value
Hemoglobin [mmol/L]			
equilibration	5.26 [4.86–5.65]	5.49 [5.25–5.72]	0.13
aorta cross-clamp	4.40 [3.92–4.88]	4.22 [3.83–4.61]	0.52
60 min cardiac arrest	6.16 [5.81–6.50]	5.98 [5.56–6.39]	0.46
120 min recovery	5.89 [5.24–6.54]	5.89 [4.83–6.82]	0.90
Hematokrit			
equilibration	25.9 [23.9–27.9]	27.1 [26.0–28.3]	0.12
aorta cross-clamp	21.7 [19.3–24.2]	20.9 [18.9–22.8]	0.26
60 min cardiac arrest	29.9 [28.6–31.2]	29.6 [27.5–31.6]	0.37
120 min recovery	29.1 [25.9–32.3]	28.8 [23.8–33.7]	0.45
lactate [mmol/L]			
equilibration	1.28 [1.02–1.54]	1.01 [0.83–1.19]	0.03
aorta cross-clamp	1.60 [1.23–1.97]	1.81 [1.15–2.47]	0.26
60 min cardiac arrest	6.26 [4.34–8.17]	2.64 [1.45–3.84]	<0.01
120 min recovery	3.71 [2.90–4.53]	2.40 [1.25–3.55]	0.03
pH value			
equilibration	7.35 [7.28–7.42]	7.36 [7.31–7.42]	0.38
aorta cross-clamp	7.38 [7.29–7.46]	7.36 [7.27–7.45]	0.40
60 min cardiac arrest	7.15 [7.04–7.26]	7.31 [7.22–7.39]	<0.01
120 min recovery	7.25 [7.17–7.33]	7.29 [7.26–7.32]	0.16
sodium [mmol/L]			
equilibration	137 [134–141]	139 [137–140]	0.26
aorta cross-clamp	135 [132–139]	137 [136–138]	0.08
60 min cardiac arrest	137 [132–142]	140 [139–142]	0.09
120 min recovery	137 [132–142]	139 [135–143]	0.24
chloride [mmol/L]			
equilibration	107 [103–110]	108 [106–110]	0.23
aorta cross-clamp	110 [108–113]	111 [109–112]	0.41
60 min cardiac arrest	107 [104–111]	110 [108–112]	0.06
120 min recovery	110 [106–113]	112 [109–114]	0.18
calcium [mmol/L]			
equilibration	1.36 [1.28–1.44]	1.37 [1.34–1.41]	0.35
aorta cross-clamp	1.38 [1.31–1.45]	1.41 [1.36–1.45]	0.23
60 min cardiac arrest	1.35 [1.28–1.42]	1.40 [1.30–1.50]	0.18
120 min recovery	1.35 [1.24–1.46]	1.49 [1.33–1.65]	0.06
potassium [mmol/L]			
equilibration	4.44 [4.11–4.78]	4.16 [3.81–4.50]	0.09
aorta cross-clamp	7.76 [6.16–9.35]	6.43 [5.53–7.34]	0.06
60 min cardiac arrest	5.83 [4.88–6.79]	4.91 [4.51–5.31]	0.03
120 min recovery	5.02 [4.40–5.64]	4.79 [4.17–5.40]	0.27

Values shown as mean [95% confidence interval]. SACP, selective anterograde cerebral perfusion; SACP + DP, selective anterograde cerebral perfusion with additional distal perfusion.

**Table 3:** Morphometric analyses of intestinal and colonic structures. Measurements in µm.

Tissue	Parameter	SACP (*n* = 9)	SACP + DP (*n* = 9)	*p* Value
small intestine	villi height	225.7 [190.8–260.7]	254.3 [206.4–302.1]	0.15
	villi width	94.9 [87.6–102.2]	99.3 [93.2–105.4]	0.15
	crypts depth	137.6 [117.3–157.9]	141.5 [126.0–156.9]	0.36
	crypts width	32.7 [29.4–35.9]	32.9 [27.8–38.0]	1.00
colon	enterocyte height	17.22 [14.3–20.2]	15.2 [13.0–17.3]	0.37
	crypts depth	294.1 [233.6–354.6]	228.4 [194.1–262.8]	0.02
	crypts width	55.2 [41.3–69.0]	49.1 [44.1–54.0]	0.67
colonic layers	mucosa	339.0 [291.9–386.2]	298.4 [268.4–328.5]	0.05
	submucosa	211.5 [119.8–303.2]	251.7 [168.4–335.0]	0.23
	muscularis propria	255.8 [160.2–351.4]	304.3 [211.6–397.0]	0.24

Values shown as mean [95% confidence interval]. SACP, selective anterograde cerebral perfusion; SACP + DP, selective anterograde cerebral perfusion with additional distal perfusion.

**Table 4:** Classification score of ischemic mucosal damage in the small intestine.

Groups	Grade 0	Grade 1	Grade 2	Grade 3	Grade 4	Grade 5
SACP (*n* = 9)	0 (%)	0 (%)	2 (22%)	1 (11%)	3 (33%)	2 (22%)
SACP + DP (*n* = 9)	1 (11%)	0 (%)	1 (11%)	5 (55%)	2 (22%)	0 (%)

SACP, selective anterograde cerebral perfusion; SACP + DP, selective anterograde cerebral perfusion with additional distal perfusion.

**Table 5:** Pathohistological analyses of small intestinal structures.

Parameter	SACP (*n* = 9)	SACP + DP (*n* = 9)	*p* Value
tissue erosion			0.05
no	2 (22%)	2 (22%)	
low grade	0 (0%)	4 (44%)	
moderate grade	0 (0%)	1 (11%)	
high grade	7 (78%)	2 (22%)	
hyperemia			0.09
no	0 (0%)	1 (11%)	
low grade	2 (22%)	4 (44%)	
moderate grade	0 (0%)	2 (22%)	
high grade	7 (78%)	2 (22%)	
edema			0.77
no	1 (11%)	0 (0%)	
low grade	3 (33%)	3 (33%)	
moderate grade	3 (33%)	4 (44%)	
high grade	2 (22%)	2 (22%)	
villus stunting			0.39
no	1 (11%)	2 (22%)	
low grade	5 (56%)	3 (33%)	
moderate grade	0 (0%)	2 (22%)	
high grade	3 (33%)	2 (22%)	

SACP, selective anterograde cerebral perfusion; SACP + DP, selective anterograde cerebral perfusion with additional distal perfusion.

**Table 6:** mRNA expression of HIF-1α, NRF2, DJ-1, E-cadherin, claudin-1, and occludin in the SACP and SACP + DP group. Data represented ∆Ct values of targets and RPL4.

Parameter	Tissue	SACP (*n* = 9)	SACP + DP (*n* = 9)	*p*-Value
HIF-1α	small intestine	4.2 [3.4–5.1]	3.7 [3.1–4.3]	0.12
	colon	3.1 [2.7–3.5]	3.4 [3.0–3.8]	0.09
NRF2	small intestine	3.1 [2.3–3.9]	2.3 [1.6–2.9]	0.04
	colon	2.4 [1.8–2.9]	2.1 [1.6–2.6]	0.19
DJ-1	small intestine	8.4 [7.2–9.6]	6.9 [6.2–7.6]	0.02
	colon	7.5 [6.5–8.5]	7.2 [6.7–7.7]	0.27
E-cadherin	small intestine	2.0 [0.89–3.1]	1.6 [0.7–2.5]	0.29
	colon	0.1 [−0.4–0.6]	0.7 [0.3–1.2]	0.04
claudin-1	small intestine	11.3 [9.4–13.1]	10.7 [9.3–12.0]	0.27
	colon	9.9 [7.8–12.1]	9.0 [8.1–9.9]	0.18
occludin	small intestine	4.5 [3.7–5.3]	4.2 [3.1–5.3]	0.80
	colon	4.5 [3.8–5.1]	4.4 [3.7–5.0]	0.40

Values shown as mean of ∆CT values [95% confidence interval]. Low ∆CT values indicate higher expression of the target mRNA being analyzed, while higher ∆CT values indicate lower expression. DJ-1, Parkinson’s disease protein; HIF-1α, hypoxia-induced factor-1α; NRF2, nuclear factor erythroid 2-related factor 2; RPL4, ribosomal protein L4; SACP, selective anterograde cerebral perfusion; SACP + DP, selective anterograde cerebral perfusion with additional distal perfusion.

**Table 7:** Oxidative stress in intestinal tissue. Measurements of enzyme activity for nicotinamide-adenine dinucleotide phosphate oxidase (NOX), superoxide dismutase (SOD), and catalase.

Parameter	Tissue	SACP (*n* = 9)	SACP + DP (*n* = 9)	*p*-Value
NOX [∆µU/mg]	small intestine	13.4 [8.7–18.2]	11.4 [4.7–18.1]	0.29
	colon	16.4 [13.0–19.8]	14.6 [11.9–17.4]	0.18
SOD [∆µU/mg]	small intestine	0.03 [0.00–0.05]	0.01 [−0.01–0.03]	0.30
	colon	0.15 [0.13–0.17]	0.14 [0.09–0.18]	0.55
Catalase [∆µU/mg]	small intestine	30.8 [26.8–34.8]	28.1 [21.8–34.5]	0.21
	colon	37.8 [31.0–44.5]	36.7 [31.8–41.6]	0.80

Values shown as mean [95% confidence interval]. NOX, nicotinamide-adenine dinucleotide phosphate oxidase; SACP, selective anterograde cerebral perfusion; SACP + DP, selective anterograde cerebral perfusion with additional distal perfusion; SOD, superoxide dismutase.

## Data Availability

Data available on request due to restrictions.

## Abbreviations

The following abbreviations are used in this manuscript:

AIF	apoptosis-inducing factor
CO	cardiac output
CPB	cardiopulmonary bypass
CVP	central venous pressure
DJ-1	Parkinson’s disease protein
HIF-1α	hypoxia-induced factor-1α
HR	heart rate
IR	ischemia-reperfusion
MAP	mean arterial pressure
NOX	NADPH oxidase
NRF2	nuclear factor erythroid 2-related factor 2
PBS	phosphate-buffered saline
ROS	reactive oxygen species
RRsys/dias	systolic/diastolic blood pressure
SACP	selective anterograde cerebral perfusion
SACP + DP	SACP with additional distal perfusion
SOD	superoxide dismutase

## Appendix A

### Appendix A.1. Animals and Anesthesia Management

For this study, one- to four-week-old, healthy suckling German Landrace/hybrid with Peitrain piglets (2.5–10 kg) of both sexes were randomly assigned to the treatment groups SACP (*n* = 9) and SACP + DP (*n* = 9). A third group was used to evaluate the effects of hypothermia, CPB and cardioplegia. All piglets were included in the analysis. In addition, 11 pigs weighing 50–90 kg were used for blood donation. A cross-reaction with donor blood was performed prior to surgery to minimize the risk of a transfusion reaction. Piglets were allowed to suckle and had ad libitum access to drinking water until the time of premedication. To avoid causing unnecessary pain or stress through restraint during venous catheter insertion, piglets were sedated (midazolam 0.5–1 mg/kg, atropine 0.02 mg/kg, ketamine 22 mg/kg i.m.). Before intubation, the anesthesia was deepened further with propofol boluses (1–10 mg/kg i.v.), and a continuous infusion of fentanyl (1–4 µg/kg Bolus + 35 µg/kg/h i.v.) was administered via the lateral auricular vein. Additionally, piglets received an intramuscular injection of 50 mg/kg metamizole before the surgical procedure. To maintain a stable body temperature, the piglets were actively warmed outside the hypothermia chamber during transportation. A single piglet was operated on per experimental day. Anaesthesia was maintained by intravenous application of 8–16 mg/kg/h propofol and 35 µg/kg/h fentanyl. The depth of anesthesia was checked before the surgical incision by means of the interdigital reflex on the hind limb (flexor reflex, supramaximal stimulus) and adjusted if necessary. If an animal showed a sudden increase or decrease in blood pressure or an increase in heart rate as a result of a painful stimulus, the anesthesia was subsequently deepened further. Piglets were orally intubated in prone position using a 3–5 mm tube and mechanically ventilated (IPPV, Hamilton Medical, Bonaduz, Switzerland) with an initial respiratory rate between 30–60/min, tidal volume of 6–8 mL/kg bodyweight, inspiratory to expiratory time 1:1.7, PEEP 5 mbar, peak pressure 17–25 mbar, and 21–50 Vol% oxygen. The piglets were fitted with a pulse oximeter at the left foreleg. Ventilation parameters were adjusted according to a peripheral oxygen saturation of >85% and blood gas analysis (ABL 90flex, Radiometer GmbH, Krefeld, Germany). Regional oxygenation of the lower abdomen and cerebrum was continuously measured using neonatal OxyAlert™ NIRSensors (INVOS-Oxymeter, Covidien Medtronic, Neustadt an der Donau, Germany), applied to the skin at the thoracal and lumbar level and bilateral at the forehead using an INVOS Oximeter 5100C (Covidien, Germany). An adhesive neutral electrode (NESSY^®^Plate 70, Erbe USA, Marietta, GA, USA) was stuck to the abdomen. Fluid substitution was initiated using a whole electrolyte solution (Jonosteril^®^, Fresenius Kabi, Germany) of 5–15 mL/kg/h by an infusion set with a flow regulator (Polytrol Set, Polymed Medical Devices, Brussels, Belgium). A three-lumen central catheter (CVC, 4F × 8cm, Arrow^®^, Teleflex Medical, Limerick, Ireland) was inserted into the right external jugular vein for anesthetic, medical, and fluid administration, as well as central venous pressure measurements and calibration of the PiCCO system (PULSION Medical Systems, Feldkirchen, Germany). An additional catheter (4F × 8 cm, Arrow^®^, Teleflex Medical, Ireland) was placed in the V. cava caudalis via V. iliaca for blood collection. Arterial catheters were inserted into the groin or femoral artery (PiCCO, 5F, PULSION Medical Systems) and the carotid artery (22 G × 5 cm; Arrow^®^, Teleflex Medical, Ireland) to measure cranial and caudal arterial blood pressure and cardiac output (CO) and systemic vascular resistance index (SVRI) as well as to collect arterial blood samples. A dose of 500–1000 IU of heparin was administered to increase the activated clotting time (ACT) to >400 s. Analyses of blood gases, lactate, and ACT were documented every 30 min and every 10 min during cardiac ischemia and reperfusion. Heart rate, blood pressure, oxygen saturation, body temperature, and ventilation parameters were monitored.

All experimental procedures were carried out under deep anesthesia with continuous physiological monitoring and were followed by euthanasia under anesthesia to minimize stress and pain. To optimize the scientific validity while minimizing animal suffering, we defined the following endpoints: untreatable drop in blood pressure, uncontrollable bleeding and perforation of heart structures. The application of three pilot animals was established to transfer the techniques from babies to piglets, to randomize the experimental groups, to blind the evaluating laboratory staff, and to ensure high reproducibility, high significance, and high transferability to the clinic.

### Appendix A.2. Randomization and Blinding

A simple randomization was performed before each operation day guaranteeing an unpredictable allocation sequence of the two different perfusion strategies. The laboratory technicians and staff involved in data analysis and interpretation were blinded until the end of the molecular biological, protein biochemical, histological and statistical analyses and were not involved in the randomization process.

### Appendix A.3. Blood and Tissue Sampling

Blood was withdrawn from the carotid artery, external jugular, and caudal cava vein after successful cannulation of vessels at equilibration, at the beginning and the end of cardiac arrest, and after 120 min of recovery. Additional blood samples were collected during 60 min of cardiac arrest. Lactate concentrations, pH values, and electrolyte concentrations were quantified using an ABL 90flex (Radiometer). Serum samples were collected during equilibration and after 120 min of recovery. After withdrawal, samples were centrifuged at 2000× *g* for 10 min at RT, aliquoted, and frozen at −20 °C. Following the euthanasia of the piglets, intestinal biopsies were immediately extracted from the small intestines at the distal end of the ileum and of the colon. All specimens were rinsed with cold 0.9% NaCl to remove digestive components and cut into 5 mm thick biopsies. Ten randomly collected tissue biopsies were snap-frozen in liquid nitrogen and stored at −80 °C from each intestinal region for molecular analyses. For histological analyses, five randomly collected tissue biopsies were fixed in 4% formaldehyde/phosphate-buffered saline (PBS), pH 7.4 for 36–48 h. Following fixation, the intestinal regions were embedded in paraffin for histological analyses.

### Appendix A.4. RNA Isolation and Quantitative Real-Time PCR (RT-qPCR)

RNA was isolated from ≤ 100 mg of snap-frozen intestinal tissue followed by cDNA synthesis as described previously [45]. For RT-qPCR, the SYBR^®^ Green PCR Master Mix (Life Technologies, Carlsbad, CA, USA) was utilized to quantify mRNA levels of Parkinson’s disease protein (DJ-1), nuclear factor erythroid 2-related factor 2 (NRF2), claudin-1 (CLDN1), occludin (OCDN) and cadherin-1 (CDH1/E-cadherin) hypoxia-induced factor-1α (HIF-1α) according to the manufacturer’s instructions. Primer sequences are shown in Table A1. Data were normalized to ribosomal protein L4 (RPL4) expression. ΔCt values were used to calculate relative expression levels.

**Table A1:** Primer sequences for RT-qPCR analysis.

Target Gene	Forward Primer 5′-3′	Reverse Primer 5′-3′
*HIF-1α*	CACACAGAAATGGCCTTGTGAA	TGTTCATAGTTCTCCCCCTGC
*DJ-1*	AAGTTACGACGCACCCACTT	GCAAACTCGAAGCTGGTTCC
*CDH1*	CCCCAACACTTCTCCCTTCA	ACTCGAGGGTTTTCTTTGGCT
*NRF2*	AGGTAGACGACATGCAACAGG	TGGACTTGGAACCGTGCTAG
*CLDN1*	TCATTGACACTGAGATCTTCGACT	TGAAAATGGCTTCCCTCCTGT
*OCDN*	CTGTGAAAACTCGAAGCAAGATGT	GAGGAGGCATGTCTTGGGTG
*RPL4*	AACCCGTATGCAAAGACAATG	ACCCCCTTCTCACCTGATTT

CDH1, cadherin-1; CLDN1, claudin-1; DJ-1, Parkinson’s disease protein; NRF2, nuclear factor erythroid 2-related factor 2; OCDN, occludin; HIF-1α, hypoxia-induced factor-1α; RPL4, ribosomal protein L4.

### Appendix A.5. Enzyme Activity Measurements

The enzyme activity of the ROS-producing enzyme NOX and ROS-degrading enzymes, catalase and SOD, were measured in protein extracts of intestinal tissue biopsies. To determine the enzyme activity, snap-frozen tissue (≤100 mg) samples were homogenized in 500 µL of a lysis-relaxing buffer (90 mM HEPES, 126 mM KCL, 36 mM NaCl, 1 mM MgCl_2_, 50 mM EGTA, 8 mM ATP, 10 mM creatine-phosphate) and a 1× Protease/Phosphatase Inhibitor Cocktail (Thermo Fisher Scientific Inc., Waltham, MA, USA)). Tissue lysates were sonicated two times for 5 min including a 5 min cooling step on ice between the sonication cycles. Then, lysates were centrifuged for 10 min at 14,000× *g* at 4 °C. The lysate supernatant was used for further analyses, and the pellet was discarded. The protein concentrations were determined using the Pierce Microplate BCA Protein Assay Kit (Thermo Fisher Scientific Inc.) according to the manufacturer’s instructions. All tissue lysates were standardized to a protein concentration of 1 mg/mL by dilution with a lysis-relaxing buffer before further measurements of enzyme activity.

To determine NOX activity, the tissue lysates were diluted 1:40 with a Krebs Henseleit buffer (118 mM NaCl, 4.7 mM KCl, 1.6 mM CaCl_2_, 1.2 mM MgSO_4_, 1.2 mM KH_2_PO_4_, 25 mM D-glucose, 1.2 mM NaHCO_3_) containing 4 mM cytochrome C and 10 mM NADPH. Then, 150 µL of diluted tissue lysates were transferred in duplicate to a 96-well plate. Absorption at 550 nm was measured every 30 sec for 10 min using the microplate reader Infinite™ 200 PRO and i-control™ software (Tecan Group AG, Männedorf, Switzerland).

To determine catalase activity, tissue lysates were diluted 1:35 with 0.05% H_2_O_2_/PBS solution before transferring 150 µL in duplicate to a 96-well plate. Absorption at 240 nm was measured every 30 sec for 10 min.

To determine SOD activity, tissue lysates were diluted 1:20 with an activation buffer containing pyrogallol and catalase. Then, 150 µL of diluted tissue lysates were transferred in duplicate to a 96-well plate. Absorption at 420 nm was measured every 30 sec for 10 min.

### Appendix A.7. Western Blot Analysis

Intestinal tissue samples were homogenized in lysis-relaxing buffer and prepared as described in the section, Enzyme Activity Measurements. For Western Blot analyses, 30 µg of protein was separated by SDS-PAGE at 100 V using a 10% sodium dodecyl sulfate-polyacrylamide gel. After transfer onto polyvinylidene fluoride membranes (Carl Roth, Karlsruhe, Germany) at 400 mA and blocking for 1 h with 5% bovine serum albumin (Sigma-Aldrich, St. Louis, MO, USA) in Tris-buffered saline (TBS: 50 mM Tris and 500 mM NaCl), the membranes were incubated overnight at 4 °C with a specific primary antibody against full-length E-Cadherin (#3195, Cell Signaling, Danvers, MA, USA). After washing three times with TBS-Tween20, the membranes were incubated with a Horseradish peroxidase (HRP)-conjugated secondary antibody for 1 h at room temperature. Bands were visualized by the initiation of chemiluminescence with Super Signal West Dura Extended Duration Substrate (Thermo Fisher Scientific Inc.). Densitometric analysis was performed with Fusion Vision Capt V16 Software (vwr, Radnor, PA, USA), and data were normalized to the signal intensity of glyceraldehyde-3-phosphate dehydrogenase (GAPDH) (#5G4, Hytest, Turku, Finland) (Figure A3).

### Appendix A.8. Enzyme-Linked Immunosorbent Assay (ELISA)

The release of AIF and cytochrome c from mitochondria into the cytosol as well as cytosolic NRF2 were evaluated after the extraction of cytoplasmatic proteins from snap-frozen intestinal tissue by subcellular fractioning. Tissue samples were homogenized in 500 µL cold lysis buffer (50 mM Tris, 1 mM EDTA, 150 mM NaCl, 1% Nonidet P-40, 0.25% deoxycholic acid sodium salt, 1× protease/phosphatase inhibitor cocktail (Carl Roth and Thermo Fisher Scientific Inc.)) and 1% phenylmethylsulfonylfluoride. After homogenization, the cytoplasmatic fraction was collected by centrifugation at 4 °C, 14,000× *g* for 10 min, and 20,000× *g* for 20 min and aliquoted and stored at −80 °C until the time of analysis. To determine the protein concentrations of each fraction, the Pierce Microplate BCA Protein Assay Kit (Thermo Fisher Scientific Inc.) was used according to the manufacturer’s instructions. The cytoplasmatic fraction was used to quantify oxidative stress markers by NRF2 ELISA Kit (MyBioSource, San Diego, CA, USA), AIF ELISA Kit, and the cytochrome c ELISA Kit (both antibodies-online, Aachen, Germany). The Porcine CAP1 ELISA Kit was used to measure DJ-1 protein expression (MyBioSource) in total tissue lysate samples.

All ELISAs were conducted according to the manufacturer’s instructions and measurements were recorded with the microplate reader Infinite™ 200 PRO and i-control™ software (Tecan).
